# A flexible high speed pulse chopper system for an inverted neutron time-of-flight option on backscattering spectrometers

**DOI:** 10.1038/s41598-018-31774-y

**Published:** 2018-09-11

**Authors:** Markus Appel, Bernhard Frick, Andreas Magerl

**Affiliations:** 1 Institut Laue-Langevin, 71 Avenue des Martyrs, 38000 Grenoble, France; 2 Center for Medical Physics and Technology, Friedrich-Alexander Universität Erlangen-Nürnberg, Henkestrasse 91, 91052 Erlangen, Germany

## Abstract

We present the design and simulation of a high resolution inverted time-of-flight option for a neutron spectrometer with crystal analysers in backscattering, with specific reference to the IN16B spectrometer at the Institut Laue-Langevin, Grenoble. While the conventional configuration with Si 111 crystals provides sub-μeV resolution in an energy range limited to ±30 μeV, the novel BATS option (BATS: Backscattering and Time-of-flight Spectrometer) extends the energy window to 340 μeV with only a slightly increased resolution of 1.2 μeV. Moreover, the observation window can be shifted to inelastic energy transfers. To bring this about, a novel fast chopper system with disks of large diameter and complex slit pattern is used, offering high flexibility in resolution and repetition rate. The chopper system consists out of two counter rotating disk chopper pairs. It provides 7 different pulse lengths, three pulse repetition rates up to 237 Hz and can operate with Si 111 or Si 311 crystal analysers. The latter option is a unique feature which covers a Q-range up to 3.7 Å^−1^ with a resolution of 6.8 μeV. Extensive ray-tracing simulations have been used to validate the design of the pulse chopper system, set limits on the sample size, and assess the achievable energy resolutions of the different chopper configurations.

## Introduction

Neutron spectrometers for inelastic or quasielastic scattering experiments use different techniques to reach a given energy resolution and access a specific region of the momentum vs. energy transfer space (*Q*, *ω*). Direct time-of-flight (TOF) spectrometers feature a monochromatic short pulse incident beam and a limited flight path in the secondary spectrometer. As a consequence, slow long wavelength neutrons, of up to 16 Å, are needed to achieve energy resolutions down to several μeV, which in turn limits the accessible range of elastic momentum transfer *Q*. Neutron backscattering (BS) spectrometers use Bragg angles of 90° on a crystal monochromator and analyser to achieve much higher sub-μeV resolution at shorter wavelengths, typically e.g. 6.27 Å for the Si 111 reflection, but at the cost of a restricted accessible energy transfer window. The gap between these two techniques is bridged by inverted TOF-BS spectrometers^1–6^ by taking advantage of the strong points of the former two techniques. Present designs have a long flight path in the primary spectrometer with a quasi-white, short pulsed incident beam and use a near-backscattering crystal analyser in the secondary spectrometer.

Commonly, inverted TOF spectrometers are installed at pulsed neutron sources, exploiting the high peak intensity concentrated in short bursts of the source. The BATS option (BATS: Backscattering and Time-of-flight Spectrometer) implements this concept at a continuous source and is a novelty pursued mainly for two reasons: Firstly, it allows to extend substantially the energy range of a reactor-based backscattering spectrometer and thus the experimental possibilities. Secondly, a continuous source offers more flexibility in that the pulse repetition rate of the spectrometer is not bound to the source frequency ranging typically between 14 Hz and 60 Hz for spallation neutron sources, whereas the BATS option described in this paper allows for a repetition rate of up to 237 Hz.

A first study for the BATS option was published by van Eijck *et al*.^7^ and its implementation was foreseen in the original design of the backscattering instrument IN16B at the Institute Laue-Langevin in Grenoble (France) which was commissioned in 2013 and has since served the user community as a powerful, conventional high resolution spectrometer at a reactor source^8^. In the following, we will extend the basic concept reported for BATS in ref.^7^ and present the details of a much more versatile chopper system with a variable energy resolution extending down to 1.2 μeV. The resulting design is supported by extensive ray-tracing simulations and is the base for a flexible 4-disk chopper system that was developed, built and installed over the last years. Hot commissioning of BATS is scheduled for spring 2018.

## Inverted TOF Mode and Requirements for the BATS Chopper System

**Figure 1:**
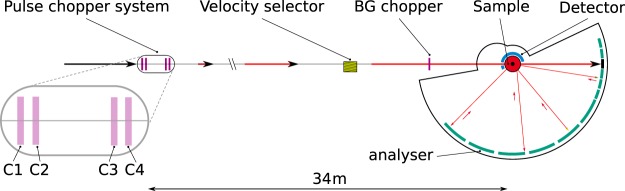
Scheme of the IN16B spectrometer in BATS configuration.

While the incident neutron energy in a conventional backscattering spectrometer is defined and modulated by a moving crystal monochromator on a Doppler drive, in BATS mode, it is determined by the different flight times of neutrons propagating from a short, quasi-white neutron pulse created by the chopper system. In both cases, the energy of neutrons scattered from the sample is analysed with crystal analysers. Figure 1 shows the IN16B BATS configuration with the beam structure created at the pulse chopper system, propagating through the wavelength band limiting neutron velocity selector to the background (BG) chopper and finally into the secondary spectrometer.

**Figure 2:**
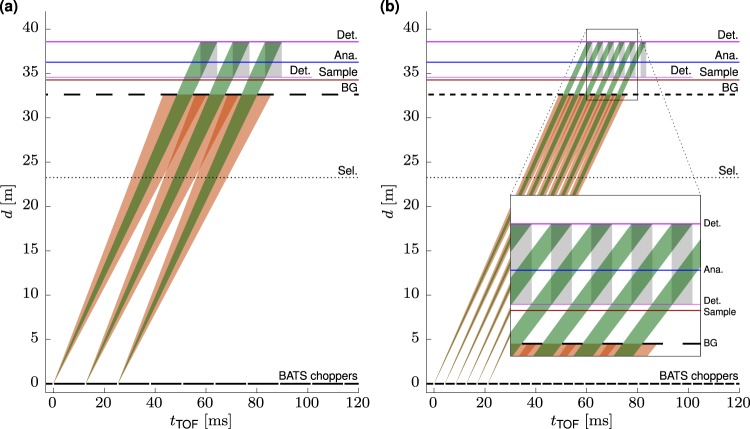
Distance vs. time diagrams of neutron pulses in the BATS configuration for the (**a**) Si 111 LRR mode and (**b**) Si 111 HRR mode.

The analyser of backscattering spectrometers like IN16B have spherical geometry with the sample in the centre to attain best energy resolution. As a consequence, the incoming neutron beam must be pulsed with a duty cycle of at most 50% to distinguish neutrons scattered into the detector with and without energy analysis at the backscattering crystals. The possible pulse repetition rates are related to the distance between sample and analyser of *d*_SA_ = 2m. With Si 111 analysers, the wavelength is *λ*_0_ = 6.271 Å corresponding to a neutron velocity of *v*_0_ = 630.8 ms^−1^. This yields a flight time from sample to analyser and back of *t*_SAS_ = 6.34 ms, which defines the maximum length of the neutron pulses at 50% duty cycle. The corresponding repetition rate is thus *f*_0_ = (2*t*_SAS_)^−1^ = 79 Hz. Figure 2a depicts this pulse structure in a time vs. distance plot. Short pulses with frequency *f*_0_ are created by the chopper system, the wavelength spectrum being restricted by the following velocity selector to a distribution with Δ*λ/λ* ≈ 12% full width half maximum (FWHM). The BG chopper operates at the same frequency *f*_0_ and transmits the central part of the spread pulse onto the sample (green), blocking out the overlapping tails of subsequent pulses (red). Note that the detector appears twice in this representation to indicate the two possible flight paths ‘Sample-Detector’ and ‘Sample-Analyser-Detector’, respectively. The time periods when energy analysed neutrons arrive on the detector are indicated by grey boxes. The width of the incident wavelength band is determined by the distance of the BATS choppers from the sample and is ideally matched to the wavelength band transmitted by the velocity selector. This results in a chopper to sample distance of *d*_CS_ = *d*_SAS_ × (Δ*v/v*)^−1^ = 33.3 m, noting that Δ*v/v* = Δ*λ/λ*.

Additionally, the secondary spectrometer allows for operation in a ‘high repetition rate’ mode (HRR) at 3*f*_0_ = 237 Hz at the expense of a reduction in the energy range as shown in Fig. 2b. Here, the analysed neutrons from each pulse are counted while the subsequent pulse has already scattered on the sample and is being reflected from the analyser. This mode allows for a threefold increase of the spectral neutron intensity on the sample due to the increased pulse frequency, but requires restriction of the wavelength band to one third yielding Δ*λ/λ* = 4% to avoid pulse overlap. As the width of the spectrum transmitted by the velocity selector cannot be changed easily, additional bandwidth choppers must be used.

The good energy resolution in a conventional BS spectrometer is mainly due to a well defined Δ*d/d* of the perfect crystal monochromator and the analysers in backscattering^9^. For the TOF-BS spectrometer BATS the secondary spectrometer comprising the analysers is identical, while the resolution of the primary spectrometer is dominated by the length of the neutron pulse created by the choppers. Ideally, the contributions of primary and secondary spectrometer are matched, which, in case of BATS, turns out to be the most critical requirement for the pulse chopper system. With an energy resolution of the secondary spectrometer with standard Si 111 analysers of *δE* = 0.6 μeV at a neutron energy of *E*_0_ = 2.08 meV, pulses as short as *δt* = 1/2 × *δE/E* × *d*_CS_/*v*_0_ = 7.6 μs are needed for a matched spectrometer. As the intensity is decreasing for shorter pulses cut from a continuous source, the chopper system should provide a reasonable set of pulse lengths to choose from. This allows to trade off intensity vs. resolution depending on the needs of the experiment to be performed.

Further, the BATS chopper system should allow for the use of Si 311 analysers in the secondary spectrometer which work with a wavelength of 3.27 Å and allow to nearly double the maximum accessible *Q* to 3.7 Å^−1^. The higher velocity of these neutrons yields a repetition rate of ${f}_{0}^{{\rm{Si}}\,{\rm{311}}}=151\,{\rm{Hz}}$ with a pulse structure similar to the ‘low repetition rate’ mode (LRR) shown in Fig. 2a.

## Pulse Chopper System Design

### Disk size, speed and slit widths

The existing neutron guide at the location of the chopper system is optimised for transporting a continuous beam for conventional backscattering with Si 111 or Si 311 crystals, i.e., for wavelength bands around 6.27 Å and 3.27 Å respectively^10^. It has thus a large, rectangular cross section of 90 mm width and 115 mm height with a reflective coating of *m* = 2, meaning that the critical angle of reflection of the walls is twice the critical angle of a simple Ni coating.

Given such large guide dimensions the circumferential velocity of the chopper disks is the most crucial aspect to attain the short pulse lengths needed, implying that both the rotation frequency and diameter of the disks should be maximised. This can be achieved by rotating the pulse chopper with a higher frequency which is an integer multiple *n* of the base frequency *f*_0_ and then using an additional suppressor chopper to skip *n* − 1 pulses. Moreover, a counter-rotating disk pair can be used to reduce the pulse length by a factor of two compared to a single disk. The diameter of the disks is subject to technical limitations, in particular since several large slits with different size are required to provide different pulse lengths for varying the energy resolution. With recent progress in the manufacturing technique of radially oriented carbon fibre reinforced polymer (oCFRP) disks^11^ it is now possible to make choppers of 750 mm diameter rotating at 4*f*_0_ = 315 Hz corresponding to a circumferential speed of 746 m s^−1^, higher than the speed of any chopper system currently in operation (see Table 1).

**Table 1:** Properties of fast turning disk choppers of selected neutron spectrometers; disk diameter *d*, rotation frequency *f*, circumferential disk speed *v*, circumferential radial acceleration *a*, disk material.

Instrument	*d*[mm]	*f*[*rpm*]	*v*[m/s]	*a*[10^6^m/s^2^]	Material	Ref.
BATS	750	19000	746	1.48	oCFRP	
TOFTOF	600	22000	691	1.59	CFRP	^15^
IN5	750	17000	668	1.19	Al	^16^
DCS	580	20000	607	1.27	Al	^17^
LET	633	18000	597	1.12	CFRP	^18, 19^

The angular opening of a slit providing full view of the guide is 20.5°. These slits create a neutron pulse length of 90 μs FWHM in case of two counter-rotating disks at 315 Hz, which is one order of magnitude longer than the 7.6 μs estimated in the previous section to match the resolution of the secondary spectrometer. This can only be attained by reducing the slit width down to 2°, which leads to a corresponding high loss of intensity due to reduction of the used guide cross section by a factor of 8. However, these losses can be mediated by adapting the neutron optics around the chopper system. The concept for an adaptive focusing of this large cross section guide to focus the neutron beam on narrower slits is discussed in ref.^12^.

In summary it is thus crucial that the BATS chopper system provides multiple slits of different widths between matched resolution (2°) and full guide view (20.5°) and includes a slower suppressor chopper to transmit only the base pulse frequency *f*_0_. As an alternative to disk choppers, a Fermi chopper (i.e. a rotating collimator) could be used but was found to be less flexible and to perform suboptimal towards shorter pulse lengths (see Supplementary Materials).

### Disk slit pattern and modes of operation

**Figure 3:**
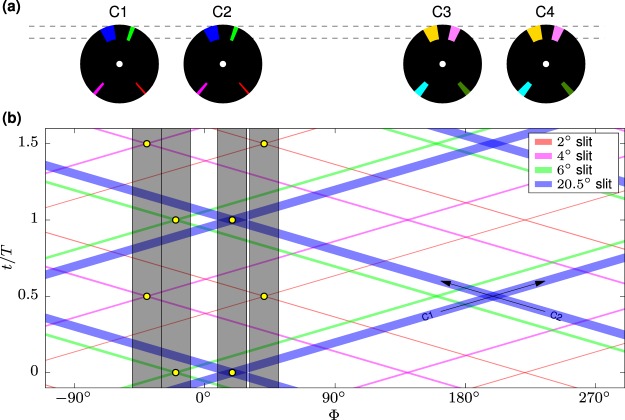
(**a**) Sketch of the slit pattern on the BATS disk chopper system; (**b**) Visualisation of overlapping slits during a full counter-rotation of the first chopper pair C1/C2. Overlapping events of corresponding slit pairs are marked with circles, the four grey boxes correspond to the guide width centered at the respective markers.

The BATS chopper system uses 4 disk choppers grouped in two counter-rotating pairs as shown in Fig. 1. They are located at 34.2 m and 33.3 m upstream of the sample, respectively, where the exact positions needed to be adjusted due to constraints imposed by neighboring beamlines. Each disk features four slits of different width: 2°, 4°, 6°, 20.5° for the first disk pair, and 8°, 11°, 14°, 20.5° for the second pair. The 20.5° opening corresponds to full guide view and is used to park the choppers in full transmission to recover the conventional backscattering mode of operation. The positions of the slits in the disks are sketched in Fig. 3a. The slit arrangement has been defined such that only one corresponding slit pair overlaps within the guide during a full rotation of each disk pair. The graph shown in Fig. 3b visualises this arrangement, showing the polar angle of each slit vs. relative time per period for the first counter-rotating disk pair. Slits on the first disk are represented by bands from bottom left to top right, while slits on the second disk run from bottom right to top left. Overlaps of corresponding slits are marked with a yellow circle. The four vertical, shaded boxes represent the width of the guide aligned on each marker respectively. In practice this is achieved by tuning the chopper phases. All spurious overlaps of non-matching slits are located outside of the guide cross section for all configurations. This representation is very helpful as it provides graphical evidence for clean measurement configurations. However, it ignores the finite distance between the chopper disks, and the divergence and wavelength distribution of the neutron beam. To avoid any spurious transmission it is thus crucial that the chopper system is examined with ray tracing simulations as discussed in the next section.

The use of two counter-rotating disk pairs with multiple slits offers great experimental flexibility and allows to provide all required modes of operation, i.e., the use of Si 111 (both LRR and HRR) as well as Si 311 analysers with variable resolution. Table 2 gives an overview of these modes with the chopper related parameters in the columns to the right. For the LRR mode with Si 111 analysers, either chopper pair can be run at 315 Hz to create short pulses while the other pair acts as suppressor chopper at a ratio of 4:1. One can choose one out of seven different slits available across both pairs (2°, 4°, 6°, 8°, 11°, 14°, 20.5°) to create the neutron pulse, providing a graduated choice in the compromise between energy resolution and intensity.

**Table 2:** Overview of different modes of operation of the BATS configuration with expected energy resolution FWHM *δE*, full width of the accessible window in energy transfer Δ*E*, accessible momentum transfer range *Q*, sensible choice of sample size with width *w*_s_ and height *h*_s_, analysed neutron energy *E*_f_, rotation frequency *f* of first (C1/C2) and second (C3/C4) chopper pair and used slit width.

	*δE* [μeV]	Δ*E* win. [μeV]	*Q* [Å^−1^]	*w*_s_ × *h*_s_ [mm^2^]	*E*_f_ [meV]	C1/C2	C3/C4
						*f* (slit width) [Hz]	*f* (slit width) [Hz]
Si 111 LRR	1.2	340	0.2 to 1.9	4 × 10	2.08	315 (**2**°)	79 (20.5°)
	2.1	340	0.2 to 1.9	4 × 20	2.08	315 (**4**°)	79 (20.5°)
	2.9	340	0.2 to 1.9	4 × 40	2.08	315 (**6**°)	79 (20.5°)
	3.9	340	0.2 to 1.9	10 × 40	2.08	79 (20.5°)	315 (**8**°)
	5.8	340	0.2 to 1.9	22 × 40	2.08	79 (20.5°)	315 (**11**°)
	7.6	340	0.2 to 1.9	22 × 40	2.08	79 (20.5°)	315 (**14**°)
	8.6	340	0.2 to 1.9	22 × 40	2.08	315 (**20.5**°)	79 (20.5°)
Si 111 HRR	1.4	120	0.2 to 1.9	4 × 10	2.08	237 (**2**°)	237 (11°)
	2.7	120	0.2 to 1.9	4 × 40	2.08	237 (**4**°)	237 (11°)
	3.6	120	0.2 to 1.9	10 × 40	2.08	237 (**6**°)	237 (8°)
Si 311	6.8	1100	0.7 to 3.7	4 × 10	7.63	302 (**2**°)	151 (20.5°)
	14	1100	0.7 to 3.7	7 × 40	7.63	302 (**4**°)	151 (20.5°)
	20	1100	0.7 to 3.7	14 × 40	7.63	302 (**6**°)	151 (20.5°)
	28	1100	0.7 to 3.7	22 × 40	7.63	151 (20.5°)	302 (**8**°)
	39	1100	0.7 to 3.7	22 × 40	7.63	151 (20.5°)	302 (**11**°)
	53	1100	0.7 to 3.7	22 × 40	7.63	151 (20.5°)	302 (**14**°)
	59	1100	0.7 to 3.7	22 × 40	7.63	302 (**20.5**°)	151 (20.5°)

The bold face indicates which slit is used to create the short neutron pulse.

The Si 111 HRR mode is realised by running both pairs at the same speed of 3*f*_0_ = 237 Hz, creating a short pulse with the first pair and limiting the transmitted wavelength band with the second pair. The slit in the second chopper pair, located downstream at a distance of 0.9 m, is chosen such that the transmitted wavelength band is narrow enough to avoid frame overlap. Therefore in this mode only three different pulse lengths can be used, but in consideration of the reduced energy transfer window only the highest resolution modes are considered relevant. For use with Si 311 analysers, the ‘low repetition rate’ modes are adapted with a slightly reduced pulse chopper frequency of $2{f}_{0}^{{\rm{Si}}\,{\rm{311}}}=302\,{\rm{Hz}}$ and a suppressor chopper ratio of 2:1 (151 Hz). The higher neutron energy selected by the Si 311 reflection leads to a broadened energy resolution *δE*, but in turn opens access to higher scattering vectors up to 3.7 Å^−1^.

## Simulation of Instrument Performance

Having multiple slits on the BATS chopper disks brings about greater flexibility, but demands a thorough verification of the design to avoid any unexpected, spurious transmission. For this purpose ray tracing simulations are an indispensable tool to complement the above analytical considerations. Building on earlier simulations of the H112 guide and IN16B as a starting point^7,10^, a modular description of the instrument using the McStas simulation package^13^ was developed. These were used to verify the slit patterns as well as to study many aspects of the instrument performance like neutron flux and energy resolution of primary and secondary spectrometer. Moreover, a future optimisation of the neutron optics has been explored. The simulation code is released in the Supplemental Materials and also available in an online repository^14^ for future development.

### Slit pattern

**Figure 4:**
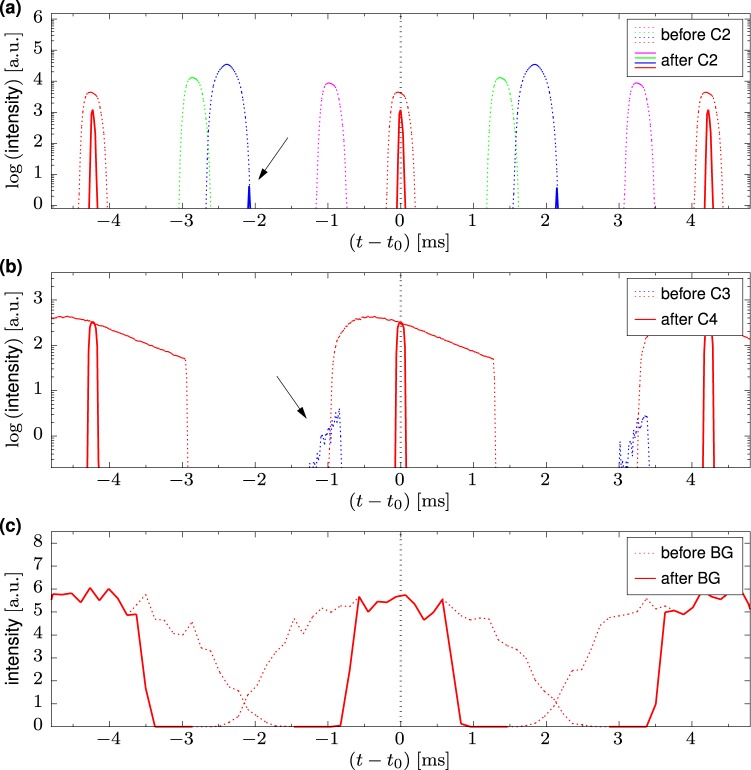
Simulated pulse transmission for the first Si 111 ‘high repetition rate’ mode in Table 2 of (**a**) the C2 chopper, (**b**) the C3/C4 chopper pair, and (**c**) the BG chopper (60° opening). Curves are color coded according to the slit color of the first chopper C1 in Fig. 3. The horizontal axis shows the time relative to the time-of-flight *t*_0_ of the central neutron wavelength at the respective instrument components. A spurious transmission of the first chopper pair is marked with the arrows; it is not transmitted by the second chopper pair.

Each of the configurations listed in Table 2 is checked to transmit pulses as intended, i.e. only through the selected slit pairs. As an example, Fig. 4a shows the pulse structure before and after the second disk of the first chopper pair for the first Si 111 HRR mode of Table 2. From the four pulses transmitted by the first disk, only the red pulse corresponding to the 2° slit is selected by the counter-rotating second disk. Some spurious transmission in the simulated wavelength band of 1 Å to 12 Å from the large 20.5° slit (blue, see arrow) of the second disk is found.

The second graph in Fig. 4b shows the pulse structure before and after the second counter-rotating chopper pair C3/C4, which selects only a narrow part of the spectrum expanded with flight time. The spurious transmission from the first pair (see arrow) has moved towards larger (*t* − *t*_0_) as it contains only neutrons from the short wavelength end of the spectrum. These are suppressed at this point as the disks of the second pair are closed during the respective time window of their arrival. If longer wavelengths than the simulated 12 Å were to be transmitted by the BATS choppers, they would be eliminated by the neutron velocity selector.

Figure 4c shows the same train of three consecutive pulses before and after the BG suppressor chopper, located 32.7 m downstream of the first chopper C1. The pulses have expanded even more, and the faster neutrons of each pulse overlap with the slower neutrons from the previous pulse. However, the overlap region falls into the time period which is suppressed by the BG chopper to attain the necessary <50% duty cycle for the secondary spectrometer as mentioned above. In this way only clean, separated pulses reach the sample position.

### Energy resolution and acceptable sample size

While the opening time of the pulse chopper discussed above is the most crucial feature determining the energy resolution of the spectrometer, there are more factors that need careful attention to ensure a realistic estimation. They all relate to the distribution of lengths of flight paths for neutron rays from the pulse chopper to the detector. The relation between the distribution of flight path length Δ*d* and energy resolution Δ*E* can be expressed as Δ*E/E* = 2Δ*d/d*, meaning that 8 mm difference in total flight path length contributes 1 μeV in energy resolution for neutrons at *E* = 2.08 meV with *d* = 34.2 m. It is thus critical to assess the contributions to the flight path distribution according to the following list:

- *Neutron guide of primary spectrometer:* The large *m* = 2 neutron guide transports neutrons of finite divergence. Thus the length of the flight path depends on the angle of the ray reflected at the guide walls. Moreover, possible future modifications of the guide for adapted focusing on smaller chopper slits and improved focusing on the sample can change this distribution and need to be taken into account.
- *Sample geometry:* It is evident that the standard sample sizes used on IN16B (flat slab of 30 × 40 mm^2^ or hollow cylinder with 22 mm diameter) are too big to attain an energy resolution below ≈5 μeV in TOF mode as they introduce large distributions of flight path lengths. Acceptable sample sizes are obtained from simulations.
- *Secondary spectrometer:* The secondary spectrometer contains spherical analyser segments that are usually aligned on a common sphere centered on the sample position. Scattered neutrons are focused back on the sample position and projected on the cylindrical detector arranged on the opposite side behind the sample (c.f. Fig. 1), which introduces large differences in the flight path (on IN16B up to 40 mm). Vertically position sensitive detector tubes allow to correct for this contribution in data reduction, leaving only an uncertainty in the detection depth. On IN16B, this corresponds to a 13 mm thick ^3^He volume, but due to its strong absorption the effective detection depth is much smaller, e.g. 50% of neutrons selected by Si 111 analysers are absorbed within the first 1.5 mm. This is also accounted for in the McStas simulations.

**Figure 5:**
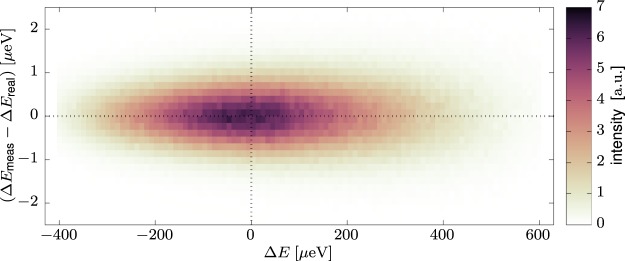
Simulated energy resolution function (Δ*E*_meas_ − Δ*E*_real_) vs. energy transfer Δ*E* for the highest resolution configuration (first line in Table 2).

With a realistic modelling of all relevant effects, the ray tracing simulation can be expected to give a reliable estimation of the final energy resolution *δE*. In the simulation, the energy resolution is not obtained as in a real experiment by using a purely elastic scattering sample as this would be computationally inefficient. Instead, an artificial sample is used which scatters each neutron ray into a fixed energy window around the energy acceptance of the analyser, e.g. the range between (2080 ± 5) μeV in case of Si 111 analysers. The energy resolution is then obtained by observing, as a function of energy transfer Δ*E*, the difference (Δ*E*_meas_ − Δ*E*_real_) which refers to the following quantities: Δ*E*_meas_ is the energy transfer as it would be measured in the spectrometer, calculated from the time-of-flight of the neutron ray from the pulse chopper to the detection point; Δ*E*_real_ is the actual energy transfer applied to the neutron ray during the simulated scattering on the artificial sample. The difference of these two values thus corresponds to the resolution of the experimental setup. As an example, the resulting distribution for the first configuration in Table 2 is shown in Fig. 5. This simulation was run with the BG chopper stopped in order to observe the full wavelength band transmitted by the velocity selector. However, in reality, the BG chopper is indispensable and thus only a limited window of Δ*E* can be observed in the experiment. In light of envisaged future modifications of the neutron guide for adapted beam focusing, we simulate both the current guide as well as the focusing configuration from ref.^12^. We find slightly different values for the resolution in both cases, most probably because a larger phase space element of the incoming beam is picked up by the sample.

**Figure 6:**
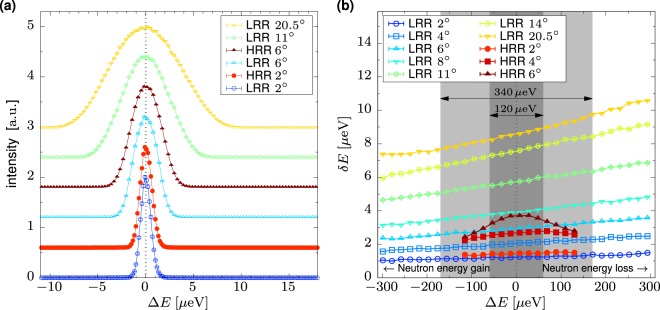
Simulated energy resolution function for Si 111 analysers with different chopper configurations of ‘low repetition rate’ (LRR) and ‘high repetition rate’ (HRR). The angle in the legend corresponds to the opening of the slit pair creating the pulse. (**a**) Elastic energy resolution function. The curves are offset vertically for clarity. (**b**) Dependence of the FWHM of the energy resolution width *δE* on energy transfer Δ*E*. The shaded areas visualize the accessible energy transfer window in case of elastic centering for LRR modes (340 μeV) and HRR modes (120 μeV).

The simulated resolution function in the elastic region for some configurations using Si 111 analysers is shown in Fig. 6a. The width of the resolution function depends on energy transfer as shown in Fig. 6b, narrowing for Δ*E* < 0 as the incident energy *E*_*i*_ is decreasing. A noticeable behavior is observed for the Si 111 HRR modes, where especially for the 6° slit a strong triangular envelope appears on top of the weak monotonous dependency such that the resolution is narrowing both for Δ*E* < 0 and Δ*E* > 0. A similar, less pronounced effect can be seen at the extremities of the curve obtained for the 4° slit. This is caused by the second chopper pair rotating at the same speed as the first pair and cutting off the wings of pulses for faster and slower neutrons, effectively narrowing the pulse width depending on neutron velocity. Note that the usable window for the HRR modes in Δ*E* is limited to a full width of at most 120 μeV, meaning that the enveloping effect will be noticeable only for the 6° slit. Moreover, if the centre of the chosen energy transfer window is offset from Δ*E* = 0, the respective chopper phases shift the envelope function to follow the centre of such an inelastic window.

From a list of all elastic resolutions for different sample geometries (see Supplementary Tables S1 to S4), a reasonable limit for the sample size of each configuration can be chosen and it is reported together with the respective energy resolution in Table 2. The simulated resolution with Si 111 analysers ranges between 1.2 μeV and 8.6 μeV with sample sizes of 4 × 10 mm^2^ for the best resolution up to the standard size of 22 × 40 mm^2^ above 5 μeV. For Si 311 analysers we expect 6.8 μeV to 59 μeV.

## Conclusion

We report on the design of a flexible pulse chopper system for an inverted TOF option on a neutron backscattering spectrometer at a continuous neutron source with specific reference to the BATS option for the instrument IN16B at the Institute Laue-Langevin. In spite of a relatively short primary flight path and a wide neutron guide at the BATS chopper location, the expected high energy resolution is made possible by recent technological progress in chopper disk construction. Two counter rotating double disk choppers each with 4 slits of different width give variable modes of operation which offer unprecedented flexibility to the experimenter. For Si 111 analysers the energy resolution can be varied in seven steps between 1.2 μeV and 8.6 μeV, ‘high repetition rate’ modes at 237 Hz provide increased flux at a reduced energy transfer range, and Si 311 analysers can be used giving access to momentum transfers *Q* up to 3.7 Å^−1^. The instrument performance was assessed by McStas ray tracing simulations, in particular to estimate the influence of sample size on the energy resolution. The simulations confirm that an inverted TOF backscattering spectrometer of the described BATS type will be most flexible and that its energy resolution will be highly competitive with any existing or planned TOF-BS spectrometer. As a significant advantage we note the possibility to realize much higher pulse repetition rates than available from spallation source frequencies. Future optimisations of the neutron optics are envisaged, allowing for adapted beam focusing on small slits of the chopper disks and improved focusing on the sample position.

## Electronic supplementary material

Supplemental Information

McStas simulation code repository
