# Supplementary material for: A flexible high speed pulse chopper system for an inverted neutron time-of-flight option on backscattering spectrometers
Source: Sci Rep. 2018 Sep 11;8:13580. doi: 10.1038/s41598-018-31774-y (PMC6133999; doi:10.1038/s41598-018-31774-y)
Supplement: Supplementary file 1 — Supplemental Information [file 41598_2018_31774_MOESM1_ESM.pdf]

Supplemental Materials: A flexible high speed pulse chopper system for an inverted neutron time-of-flight option on backscattering spectrometers

Markus Appel,[1,2,a)] Bernhard Frick,[1] and Andreas Magerl[2]

[1)] *Institut Laue-Langevin, 71 Avenue des Martyrs, 38000 Grenoble, France*

[2)] *Center for Medical Physics and Technology, Friedrich-Alexander Universität Erlangen-Nürnberg, Henkestrasse 91, 91052 Erlangen, Germany*

(Dated: 14 August 2018)

I. FERMI CHOPPER VS. DISK CHOPPER

An important aspect in the design of the chopper system is the large neutron guide cross section of $90 \times 115\,\mathrm{mm}^2$ at the position of the disks considered here. To achieve the necessary pulse lengths, the slit width of a counter rotating disk chopper pair needs to be reduced below the guide width. This means that only a reduced cross section of the guide is used and intensity is lost.

A Fermi chopper, representing a rotating collimator can take advantage of the entire guide cross section and potentially avoid this loss of intensity. For peak transmission, the collimation should not be narrower than the actual beam divergence in the neutron guide. The critical angle of reflection of 6.3 Å neutrons in a $m = 2$ guide is 1.2°. A collimator slit package with channels of 10 mm length and 0.8 mm width would be matching. The neutron pulse FWHM corresponds then to the time the rotor needs to turn by an angle of 2.4°, yielding a rotation frequency of 877 Hz for the required pulse length of 7.6 µs. For such a large rotor this is clearly beyond technical feasibility, meaning that the pulse lengths cannot be matched to the secondary spectrometer resolution. Using a narrower collimation is not helpful in this case, as the rotor still needs to traverse the full neutron beam divergence.

A quantitative assessment has been obtained by McStas simulations for Fermi choppers with different collimator slit packages. The energy resolution $\delta E_{\mathrm{prim.}}$ of the *primary spectrometer* has been analysed by observing the time structure for 6.3 Å neutrons at the sample position. We show in Fig. S1 the corresponding resolution on the abscissa, and the relative intensity per pulse divided by the resolution $I/\delta E_{\mathrm{prim.}}$ on the ordinate. The curve for the disk chopper system as described in the main part of this article is obtained by varying the slit width between 2° and 22° for a counter rotating pair at $4f_0 = 315.4\,\mathrm{Hz}$. Note, that sub-µeV resolution of the primary spectrometer is achieved with the most narrow slit pair of 2°.

The normalised intensity $I/\delta E_{\mathrm{prim.}}$ decreases linearly with smaller $\delta E_{\mathrm{prim.}}$ due to the reduced cross section of the transmitted beam for smaller slits. The curves for the Fermi chopper in Fig. S1 result from rotating the respective slit package at integer multiples of f_0 up to $6f_0 = 473.1\,\mathrm{Hz}$. Several disadvantages of the Fermi chopper become clear:

- The best achievable energy resolution is, depending on the slit package, between 1.3 µeV and 2.4 µeV which does not match to the resolution of the secondary spectrometer.

- The intensity for the Fermi chopper is for long pulses with relaxed resolution inferior to a disk chopper system.

- Because the flight time of the neutron through the slit package becomes comparable to the opening time for high frequencies of the Fermi chopper, the relative intensity is decreasing with better energy resolution. For the highest resolution it is only about a factor of 2 higher than for the disk chopper. For the disk chopper system adaptions of the neutron optics can be envisaged[S1] which will largely recover the loss of intensity, while there is hardly any possibility to improve the transmitted intensity of the Fermi chopper.

[S1]M. Appel, B. Frick, and A. Magerl, Manuscript in preparation.

[a)] E-mail: appel@ill.eu

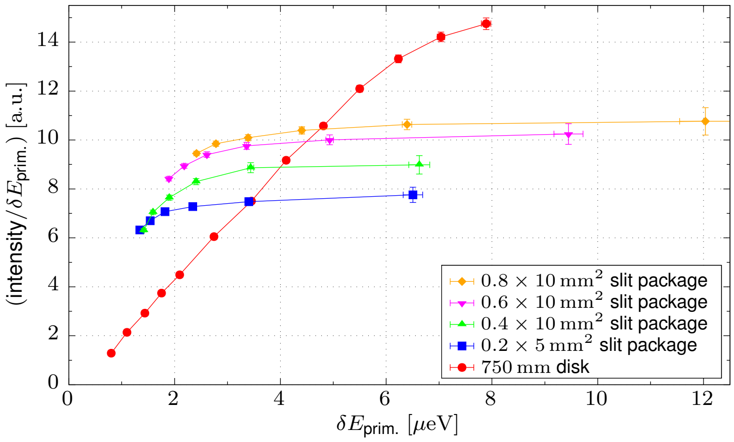

FIG. S1. Comparison of neutron intensity divided by energy resolution $\delta E_{\mathrm{prim.}}$ of the primary spectrometer transmitted by a disk chopper and a Fermi chopper with different slit packages. The curves are obtained by varying the slit width for the disk chopper and the rotation frequency for the Fermi chopper.

II. ENERGY RESOLUTION AND SAMPLE SIZE

The energy resolution in the elastic region was obtained by fitting a generalised Gaussian of the form

$$I(E) = I_0 \exp\left\{-\frac{1}{2}\left|\frac{E-E_0}{\sigma}\right|^n\right\} \tag{S1}$$

to the simulated intensity profiles similar to those shown in Fig. 6a of the main article. The obtained values for the power in the exponent were usually close to $n = 2$ for sample sizes matched to the energy resolution. The generalised form helps to obtain reliable linewidths also for unmatched combinations which lead to ‘flat-top’ shapes with $n > 2$. The full width at half maximum (FWHM) energy resolution δE was then obtained by

$$\mathrm{FWHM} = 2\sigma \sqrt[n]{2\log 2} \tag{S2}$$

and is reported in the following Tables S1 to S4 and visualised in Fig. S2.

TABLE S1. Simluated energy resolution FWHM δE in µeV using the current focusing guide in front of the sample position for flat samples oriented at a slab angle of 135°. The resolution is evaluated using the sum over all scattering angles (Σ) and using only three detector tubes under 45° and 135° scattering angle respectively. Cells are color-coded depending on their value from green (best) to red (worst).

	$4 \times 4\,\mathrm{mm}^2$ slab 135°			$4 \times 10\,\mathrm{mm}^2$ slab 135°			$4 \times 20\,\mathrm{mm}^2$ slab 135°			$10 \times 10\,\mathrm{mm}^2$ slab 135°			$4 \times 40\,\mathrm{mm}^2$ slab 135°			$10 \times 40\,\mathrm{mm}^2$ slab 135°			$15 \times 40\,\mathrm{mm}^2$ slab 135°			$30 \times 40\,\mathrm{mm}^2$ slab 135°		
configuration	Σ	45°	135°	Σ	45°	135°	Σ	45°	135°	Σ	45°	135°	Σ	45°	135°	Σ	45°	135°	Σ	45°	135°	Σ	45°	135°
Si111 lr (2° slit)	1.2	1.1	1.2	1.2	1.2	1.2	1.3	1.3	1.4	1.6	1.3	2.0	1.6	1.5	1.7	2.0	1.6	2.4	2.4	1.9	3.1	4.0	2.7	5.7
Si111 lr (4° slit)	1.9	1.8	1.8	2.0	1.9	2.0	2.0	1.9	2.0	2.1	2.1	2.3	2.2	2.2	2.3	2.5	2.3	2.6	2.8	2.4	3.2	4.2	2.9	5.8
Si111 lr (6° slit)	2.8	3.0	2.9	2.9	2.9	3.0	2.8	2.8	2.8	2.9	2.8	2.9	3.0	2.9	3.0	3.1	3.0	3.2	3.3	3.0	3.6	4.4	3.3	5.7
Si111 lr (8° slit)	4.1	3.8	4.1	4.0	4.0	4.0	4.1	4.1	4.1	4.1	4.0	4.1	4.1	4.0	4.2	4.1	4.1	4.2	4.2	4.1	4.4	5.0	4.2	5.9
Si111 lr (11° slit)	5.8	5.7	5.8	5.8	5.8	5.7	5.8	5.9	5.8	5.8	5.8	5.9	5.8	5.8	5.8	5.8	5.8	5.7	5.8	5.8	5.8	6.2	5.8	6.7
Si111 lr (14° slit)	7.9	7.7	7.8	7.7	7.6	7.7	7.6	7.7	7.6	7.7	7.8	7.7	7.7	7.7	7.7	7.7	7.7	7.7	7.8	7.8	7.8	7.8	7.7	7.9
Si111 lr (20.5° slit)	8.0	7.1	8.1	8.2	8.3	8.2	7.9	8.0	7.8	8.0	7.9	8.0	8.0	8.0	8.0	8.2	8.0	8.2	8.3	8.2	8.4	8.7	8.5	8.9
Si111 hr (2° slit)	1.4	1.4	1.4	1.4	1.3	1.5	1.5	1.5	1.6	1.7	1.5	2.1	1.8	1.7	1.9	2.1	1.8	2.4	2.5	2.0	3.1	3.9	2.7	5.6
Si111 hr (4° slit)	2.5	2.5	2.5	2.5	2.6	2.5	2.6	2.5	2.6	2.6	2.6	2.7	2.7	2.7	2.7	2.8	2.7	3.0	3.1	2.8	3.4	4.3	3.2	5.6
Si111 hr (6° slit)	3.4	3.3	3.4	3.4	3.2	3.3	3.4	3.4	3.5	3.5	3.5	3.5	3.5	3.5	3.5	3.6	3.6	3.7	3.8	3.6	4.0	4.7	3.9	5.8
Si311 (2° slit)	6.9	6.1	6.7	6.9	6.9	6.8	7.2	7.2	7.1	7.9	7.1	8.9	8.1	8.0	8.2	8.9	8.0	9.7	10.3	8.6	12.0	15.2	10.7	20.5
Si311 (4° slit)	14.3	14.7	14.8	14.0	14.1	14.0	13.7	14.0	13.6	13.9	13.7	13.7	14.1	14.1	14.2	14.4	14.2	14.6	14.8	14.4	15.4	18.0	15.0	21.8
Si311 (6° slit)	19.0	18.4	19.0	19.5	19.1	20.0	19.4	19.1	19.6	19.6	19.5	19.8	19.5	19.2	19.7	19.8	19.7	19.8	20.0	19.7	20.2	21.5	20.3	23.1
Si311 (8° slit)	28.7	28.7	29.1	28.4	28.2	28.3	28.8	28.8	28.7	28.2	28.6	27.9	29.0	29.2	28.9	28.3	28.4	28.5	28.3	28.4	28.2	28.7	27.9	29.5
Si311 (11° slit)	41.2	41.8	41.1	40.8	41.6	40.7	41.2	41.1	41.4	40.3	40.2	40.7	40.6	40.2	40.7	40.4	40.2	40.5	40.4	40.6	40.2	39.7	39.7	39.8
Si311 (14° slit)	51.9	51.1	51.2	52.9	53.4	53.0	52.5	52.7	52.1	52.8	53.4	53.0	52.5	52.1	53.0	53.1	53.2	53.3	53.5	53.5	53.5	53.3	53.5	53.4
Si311 (20.5° slit)	54.4	52.7	55.9	55.4	55.3	55.3	55.8	56.7	55.5	56.4	57.7	57.0	55.9	55.6	56.3	57.0	56.7	57.6	57.1	56.9	57.1	58.5	58.5	58.6

TABLE S2. Same as Table S1, but using a future, adapted focusing guide in front of the sample position.

	$4 \times 4\,\mathrm{mm}^2$ slab 135°			$4 \times 10\,\mathrm{mm}^2$ slab 135°			$4 \times 20\,\mathrm{mm}^2$ slab 135°			$10 \times 10\,\mathrm{mm}^2$ slab 135°			$4 \times 40\,\mathrm{mm}^2$ slab 135°			$10 \times 40\,\mathrm{mm}^2$ slab 135°			$15 \times 40\,\mathrm{mm}^2$ slab 135°			$30 \times 40\,\mathrm{mm}^2$ slab 135°		
configuration	Σ	45°	135°	Σ	45°	135°	Σ	45°	135°	Σ	45°	135°	Σ	45°	135°	Σ	45°	135°	Σ	45°	135°	Σ	45°	135°
Si111 lr (2° slit)	1.2	1.1	1.2	1.2	1.2	1.3	1.4	1.3	1.4	1.6	1.3	2.0	1.5	1.4	1.5	1.9	1.6	2.2	2.3	1.8	2.9	3.2	2.3	4.5
Si111 lr (4° slit)	2.0	2.0	2.0	2.0	2.0	2.0	2.1	2.0	2.0	2.2	2.1	2.3	2.2	2.2	2.2	2.4	2.2	2.6	2.7	2.3	3.1	3.6	2.7	4.9
Si111 lr (6° slit)	2.8	2.8	2.8	2.8	2.8	2.8	2.9	2.9	2.9	2.9	2.9	3.0	2.9	2.9	3.0	3.0	2.9	3.1	3.2	3.0	3.5	4.0	3.3	5.0
Si111 lr (8° slit)	3.8	3.7	3.9	3.9	3.8	3.8	3.9	3.9	3.9	3.8	3.8	3.9	3.9	3.9	3.9	3.9	3.9	4.0	4.0	3.9	4.2	4.7	4.1	5.4
Si111 lr (11° slit)	5.6	5.6	5.7	5.6	5.6	5.6	5.6	5.5	5.6	5.6	5.6	5.5	5.6	5.6	5.5	5.6	5.6	5.6	5.6	5.6	5.6	5.9	5.6	6.3
Si111 lr (14° slit)	7.8	7.8	7.6	7.8	7.9	7.7	7.8	7.9	7.8	7.8	7.7	7.8	7.8	7.8	7.8	7.7	7.7	7.8	7.7	7.7	7.7	7.7	7.6	7.9
Si111 lr (20.5° slit)	8.4	8.4	8.2	8.4	8.6	8.5	8.4	8.4	8.6	8.6	8.7	8.6	8.5	8.4	8.5	8.7	8.7	8.7	8.6	8.5	8.6	8.7	8.5	8.9
Si111 hr (2° slit)	1.4	1.4	1.4	1.5	1.4	1.5	1.6	1.5	1.6	1.8	1.5	2.1	1.7	1.6	1.8	2.0	1.8	2.3	2.3	1.9	2.9	3.3	2.5	4.6
Si111 hr (4° slit)	2.6	2.6	2.6	2.6	2.5	2.6	2.6	2.6	2.6	2.7	2.6	2.7	2.7	2.7	2.7	2.8	2.7	3.0	3.0	2.8	3.3	3.9	3.1	4.9
Si111 hr (6° slit)	3.5	3.5	3.5	3.5	3.5	3.4	3.5	3.4	3.5	3.5	3.4	3.6	3.5	3.5	3.5	3.6	3.5	3.7	3.7	3.6	3.9	4.4	3.8	5.2
Si311 (2° slit)	6.9	6.8	7.1	6.8	6.8	7.0	7.1	6.9	7.3	7.7	7.1	8.3	7.7	7.5	7.8	8.4	7.8	9.2	9.4	8.1	10.9	12.1	9.4	15.7
Si311 (4° slit)	14.1	14.0	14.2	14.0	13.9	14.0	14.0	13.9	14.0	14.1	14.1	14.2	14.1	14.0	14.2	14.2	14.0	14.3	14.6	14.2	15.2	16.3	14.6	18.4
Si311 (6° slit)	20.3	20.8	20.2	20.1	20.0	20.0	19.9	19.9	19.8	20.0	20.1	19.6	20.1	20.2	19.9	20.1	20.2	20.1	20.2	20.0	20.4	20.9	20.1	22.0
Si311 (8° slit)	28.0	28.4	27.8	27.9	27.8	28.3	27.9	28.0	27.9	27.5	27.7	27.9	28.0	28.0	27.9	27.7	27.7	27.5	27.7	27.6	27.7	28.1	27.7	28.5
Si311 (11° slit)	40.2	40.3	40.2	40.5	41.0	40.3	40.2	40.1	40.2	39.4	39.1	39.6	40.1	39.9	40.2	39.5	39.5	39.6	39.3	39.4	39.2	39.2	39.3	39.2
Si311 (14° slit)	54.3	54.7	53.8	54.6	54.0	54.6	54.5	54.6	54.6	53.9	54.1	53.7	54.4	54.2	54.4	54.0	54.0	54.0	53.8	53.9	53.8	53.5	53.6	53.4
Si311 (20.5° slit)	59.4	58.0	59.0	59.9	60.0	60.0	59.7	60.6	59.6	59.7	59.7	60.1	59.6	59.6	59.5	59.8	59.6	60.0	59.2	59.2	59.3	58.7	58.9	58.6

TABLE S3. Same as Table S1, but for cylindrical samples (diameter × height) using the current focusing guide in front of the sample position.

	$4 \times 10\,\mathrm{mm}^2$ cyl			$4 \times 20\,\mathrm{mm}^2$ cyl			$4 \times 40\,\mathrm{mm}^2$ cyl			$7 \times 40\,\mathrm{mm}^2$ cyl			$10 \times 40\,\mathrm{mm}^2$ cyl			$14 \times 40\,\mathrm{mm}^2$ cyl			$18 \times 40\,\mathrm{mm}^2$ cyl			$22 \times 40\,\mathrm{mm}^2$ cyl		
configuration	Σ	45°	135°	Σ	45°	135°	Σ	45°	135°	Σ	45°	135°	Σ	45°	135°	Σ	45°	135°	Σ	45°	135°	Σ	45°	135°
Si111 lr (2° slit)	1.2	1.1	1.3	1.3	1.2	1.5	1.6	1.5	1.7	1.8	1.6	2.1	2.2	1.8	2.7	2.7	1.9	3.7	3.3	2.3	4.8	4.1	2.6	5.8
Si111 lr (4° slit)	1.9	1.8	1.9	2.0	1.9	2.1	2.2	2.2	2.3	2.4	2.2	2.5	2.6	2.3	2.9	3.0	2.4	3.8	3.5	2.6	4.8	4.2	2.9	5.9
Si111 lr (6° slit)	2.9	2.7	3.0	2.8	2.8	2.7	2.9	2.9	2.8	3.0	2.9	3.1	3.1	2.9	3.4	3.4	3.1	3.9	3.8	3.2	4.9	4.4	3.3	6.1
Si111 lr (8° slit)	4.1	4.0	4.1	4.1	4.0	4.1	4.1	4.1	4.0	4.0	4.0	4.2	4.1	4.1	4.1	4.2	4.1	4.5	4.5	4.1	5.2	4.9	4.2	6.3
Si111 lr (11° slit)	5.8	5.9	5.8	5.8	5.7	5.8	5.8	5.8	5.7	5.8	5.8	5.8	5.8	5.7	5.8	5.8	5.7	5.8	5.8	5.7	5.9	5.9	5.6	6.5
Si111 lr (14° slit)	7.7	7.7	7.7	7.7	7.8	7.9	7.7	7.8	7.6	7.7	7.8	7.7	7.8	7.8	7.8	7.8	7.8	7.7	7.8	7.8	7.8	7.7	7.7	7.8
Si111 lr (20.5° slit)	7.9	7.7	7.9	7.9	7.9	8.0	8.1	8.1	8.1	8.3	8.0	8.5	8.3	8.3	8.3	8.5	8.5	8.6	8.6	8.5	8.8	8.8	8.6	8.9
Si111 hr (2° slit)	1.4	1.3	1.5	1.5	1.4	1.5	1.8	1.7	1.8	2.0	1.7	2.2	2.2	1.9	2.8	2.8	2.1	3.8	3.4	2.3	4.9	4.2	2.7	6.0
Si111 hr (4° slit)	2.5	2.4	2.6	2.6	2.5	2.6	2.7	2.7	2.7	2.8	2.7	2.9	2.9	2.7	3.2	3.2	2.8	3.9	3.7	3.0	4.9	4.3	3.1	6.1
Si111 hr (6° slit)	3.4	3.4	3.4	3.4	3.3	3.5	3.5	3.5	3.6	3.6	3.6	3.6	3.7	3.6	3.8	3.9	3.6	4.3	4.2	3.7	5.1	4.7	3.8	6.1
Si311 (2° slit)	7.0	6.6	6.9	7.3	7.1	7.4	8.1	7.7	8.4	8.5	8.0	8.8	9.4	8.4	10.7	10.8	8.8	13.6	12.8	9.5	17.8	15.3	10.3	22.0
Si311 (4° slit)	13.9	13.7	13.6	13.9	14.0	13.9	14.1	14.2	14.1	14.2	14.1	14.5	14.5	14.3	14.9	15.1	14.4	16.1	16.1	14.5	18.6	17.4	14.9	22.2
Si311 (6° slit)	19.4	19.2	18.9	19.5	19.8	19.4	19.6	19.5	19.6	19.9	19.7	20.3	19.8	19.7	20.1	20.3	20.1	20.8	20.7	20.0	21.9	21.4	20.1	24.1
Si311 (8° slit)	28.6	28.7	28.9	28.5	28.3	27.8	28.7	28.3	29.0	28.2	28.3	28.2	28.2	28.2	28.0	28.1	28.0	28.2	28.1	27.8	28.4	28.2	27.9	29.1
Si311 (11° slit)	40.9	40.6	41.0	40.9	40.7	41.6	40.5	40.6	40.0	40.6	40.6	40.6	40.4	40.5	40.1	39.8	40.1	39.4	39.2	39.2	39.1	39.1	39.1	39.3
Si311 (14° slit)	52.7	53.0	53.2	53.1	53.3	53.8	53.0	53.1	52.5	53.3	53.3	53.2	53.6	53.4	53.7	53.8	53.7	53.6	53.7	54.0	53.7	53.4	53.6	53.3
Si311 (20.5° slit)	56.8	57.0	55.9	56.3	55.9	56.2	56.5	56.3	56.3	56.7	56.4	56.5	57.2	57.3	57.5	57.6	57.4	57.8	58.4	58.3	58.7	58.6	58.5	59.2

TABLE S4. Same as Table S3, but using a future adapted focusing guide in front of the sample position.

	$4 \times 10\,\mathrm{mm}^2$ cyl			$4 \times 20\,\mathrm{mm}^2$ cyl			$4 \times 40\,\mathrm{mm}^2$ cyl			$7 \times 40\,\mathrm{mm}^2$ cyl			$10 \times 40\,\mathrm{mm}^2$ cyl			$14 \times 40\,\mathrm{mm}^2$ cyl			$18 \times 40\,\mathrm{mm}^2$ cyl			$22 \times 40\,\mathrm{mm}^2$ cyl		
configuration	Σ	45°	135°	Σ	45°	135°	Σ	45°	135°	Σ	45°	135°	Σ	45°	135°	Σ	45°	135°	Σ	45°	135°	Σ	45°	135°
Si111 lr (2° slit)	1.2	1.2	1.3	1.4	1.3	1.4	1.5	1.4	1.6	1.7	1.5	2.0	2.1	1.6	2.7	2.7	1.9	3.8	3.4	2.1	4.9	4.2	2.4	6.0
Si111 lr (4° slit)	2.0	2.0	2.1	2.1	2.0	2.1	2.2	2.1	2.2	2.3	2.2	2.4	2.5	2.3	2.8	2.9	2.4	3.8	3.5	2.5	5.0	4.3	2.7	6.3
Si111 lr (6° slit)	2.8	2.9	2.8	2.9	2.9	2.9	2.9	2.9	3.0	3.0	2.9	3.1	3.1	3.0	3.3	3.4	3.1	3.9	3.8	3.1	5.1	4.4	3.2	6.4
Si111 lr (8° slit)	3.8	3.9	3.8	3.9	3.9	3.9	3.9	3.9	3.9	3.9	3.8	3.9	3.9	3.9	4.0	4.1	3.9	4.4	4.4	3.9	5.3	4.8	4.0	6.6
Si111 lr (11° slit)	5.6	5.5	5.6	5.6	5.6	5.7	5.6	5.5	5.6	5.6	5.6	5.5	5.6	5.5	5.6	5.5	5.5	5.6	5.6	5.5	5.9	5.8	5.4	6.5
Si111 lr (14° slit)	7.8	7.8	7.7	7.8	7.8	7.8	7.8	7.8	7.8	7.8	7.8	7.7	7.7	7.7	7.7	7.7	7.7	7.6	7.6	7.6	7.6	7.6	7.6	7.7
Si111 lr (20.5° slit)	8.6	8.5	8.4	8.4	8.5	8.3	8.5	8.5	8.3	8.7	8.6	8.7	8.5	8.4	8.5	8.6	8.4	8.7	8.6	8.4	8.7	8.6	8.4	8.9
Si111 hr (2° slit)	1.4	1.4	1.5	1.6	1.5	1.6	1.7	1.7	1.8	1.9	1.7	2.1	2.2	1.8	2.8	2.8	2.0	3.9	3.5	2.3	5.0	4.3	2.5	6.1
Si111 hr (4° slit)	2.6	2.6	2.6	2.6	2.6	2.7	2.7	2.6	2.7	2.8	2.7	2.9	2.9	2.7	3.2	3.2	2.8	3.9	3.7	2.9	5.1	4.4	3.0	6.4
Si111 hr (6° slit)	3.5	3.5	3.5	3.5	3.5	3.5	3.5	3.5	3.5	3.5	3.5	3.6	3.6	3.5	3.7	3.8	3.6	4.2	4.2	3.7	5.2	4.7	3.7	6.5
Si311 (2° slit)	6.8	6.6	6.9	7.2	7.1	7.4	7.7	7.5	7.8	8.2	7.7	8.8	9.0	7.9	10.5	10.6	8.4	14.4	13.0	8.9	19.0	15.8	9.7	23.2
Si311 (4° slit)	13.9	14.2	13.7	14.0	14.0	14.1	14.0	14.0	14.2	14.2	14.1	14.4	14.4	14.2	14.8	15.0	14.3	16.2	16.0	14.4	19.4	17.6	14.5	24.2
Si311 (6° slit)	20.0	19.9	20.0	20.0	19.8	20.2	20.0	20.0	19.9	20.1	20.0	20.0	20.2	20.1	20.2	20.3	20.1	20.7	20.5	20.0	21.8	21.4	19.9	24.8
Si311 (8° slit)	28.0	28.1	28.0	28.0	27.8	27.9	28.0	28.2	27.8	27.6	27.6	27.6	27.5	27.6	27.4	27.8	27.8	27.7	27.8	27.7	28.2	28.0	27.6	29.3
Si311 (11° slit)	40.1	40.1	40.0	40.0	39.8	39.9	40.0	40.0	40.0	39.4	39.7	39.5	39.2	39.3	39.2	39.1	39.2	38.7	38.8	39.0	38.7	38.7	39.0	38.8
Si311 (14° slit)	54.5	54.7	54.2	54.7	55.1	54.6	54.5	54.2	54.5	54.0	54.1	53.7	53.6	53.7	53.6	53.6	53.7	53.6	53.4	53.6	53.3	53.3	53.6	52.8
Si311 (20.5° slit)	59.8	59.1	59.7	60.1	60.1	60.0	59.9	59.7	60.2	59.7	59.6	59.6	59.0	59.0	59.1	58.4	57.9	58.6	58.4	58.2	58.5	58.7	58.5	59.0

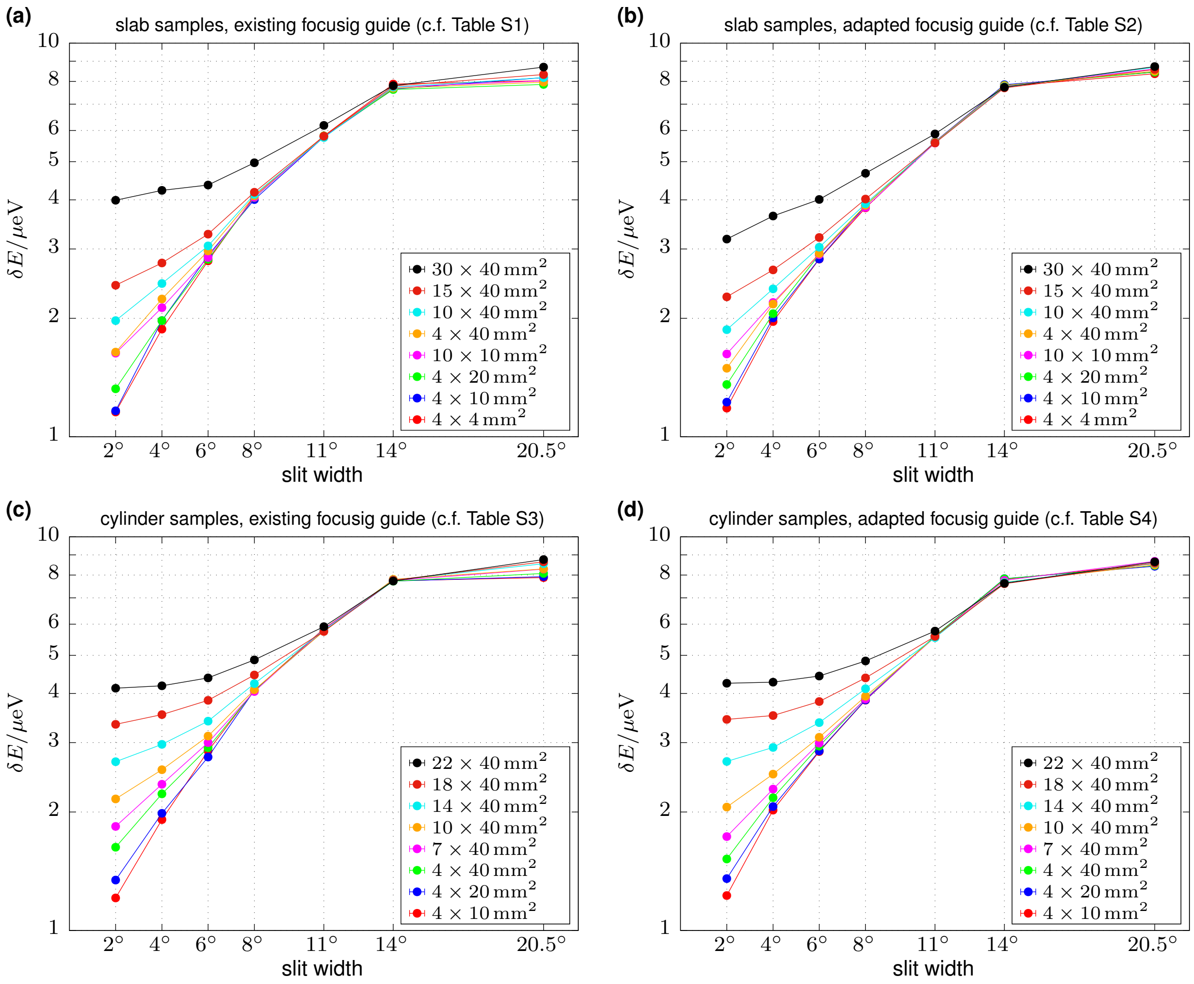

FIG. S2. Visualisation of data listed in Tables S1 to S4 of energy resolution for summed scattering angles of Si 111 lr modes.
